# Health literacy on COVID-19 and COVID-19 vaccinations in Indonesia

**DOI:** 10.12688/f1000research.125551.2

**Published:** 2022-12-23

**Authors:** Viskasari P. Kalanjati, Nurina Hasanatuludhhiyah, Annette d'Arqom, Azlin Muhammad, Ancah Caesarina Novi Marchianti, Danial Habri Arsyi, Putu Bagus Dharma Permana, I Made Dwi Yudiartana Putra Susila, Octaviana Galuh Pratiwi, Diana Purwitasari

**Affiliations:** 1 Department of Anatomy, Histology and Pharmacology, Faculty of Medicine Universitas Airlangga, Surabaya, Indonesia; 2 Faculty of Medicine, Universiti Kebangsaan Malaysia, Cheras, Kuala Lumpur, 56000, Malaysia; 3 Public Health Division, Faculty of Medicine Universitas Jember, Kalimantan Street No. 37, Kampus Tegalboto, Jember, East Java, 68121, Indonesia; 4 Faculty of Medicine, Universitas Airlangga, Street no. 47, Surabaya, East Java, 60132, Indonesia; 5 Department of Informatics, Institut Teknologi Sepuluh Nopember, Teknik Informatika Department Building, Sukolilo, Surabaya, 60111, Indonesia

**Keywords:** COVID-19, health risk, vaccines literacy, adult vaccinations, Indonesia

## Abstract

**Introduction: **Health literacy on the coronavirus disease 2019 (COVID-19) affects people’s capability to ascertain their health and health care quality during the pandemic. The objective of this study was to determine the levels of health literacy about COVID-19 vaccines and vaccinations (Vaccines and Vaccinations literacy-VL) in the Indonesian adult general population, assessing the perceptions of the respondents about current adult immunization and beliefs about vaccinations in general, and analyzing correlations of these variables with the VL levels.

**Methods: **A cross-sectional study using a rapid survey was administered via the Internet. Data were analyzed using descriptive and inferential statistics; the internal consistency of the VL scales was evaluated using Cronbach’s alpha coefficient; the inter-correlation between the functional and interactive-critical VL questions, the underlying components (factors) and each question’s load on the components were identified using a Principal Component Analysis (PCA). An alpha level lesser than 0.05 was considered significant.

**Results: **Responses to functional- and interactive/ critical- VL questions were acceptable and showed internal consistency (Cronbach’s alpha = 0.817 and 0.699, respectively), lowest values observed were 0.806 for functional scale and 0.640 for the interactive-critical scale. The PCA demonstrated that there were two components accounting for 52.45% of the total variability. Approximately 60% of respondents were females (n=686). Almost all respondents used the internet to seek information regarding COVID-19 and COVID-19 vaccinations. Many used at least one social media actively with 74.4% of respondents sometimes believing the validity of this information.

**Conclusions: **High scores were observed in both functional- and interactive/ critical-VL, and were quite in a balance between sexes in the prior VL and higher in females for the latter; these were also closely related to the educational level and age group. It is crucial to increase public health literacy in managing the pandemic.

## Introduction

Since the COVID-19 pandemic, the spread of vast information on this topic has increased, including vaccines and vaccinations. ^1^^,^^2^ One must filter this information wisely to avoid fake news that might compromise the acceptance towards the COVID-19 vaccines and vaccinations. ^3^ On the other hand, literacy levels on these subjects will also affect the opinion and personal beliefs when facing the issue. ^4^ In the ongoing development of COVID-19 vaccinations and vaccines, evidence-based data released in a real-time fashion may lead to conflict when comprehended with no further authorized confirmation. ^5^^–^^7^ These data are valuable to the decision-makers party to understand public sentiment and thus act accordingly to contain the pandemic. ^6^ The current study aimed to determine and compare public opinion and sentiments on COVID-19, COVID-19 vaccines, and vaccinations before and after the national vaccinations program was held in Indonesia (in January 2021). These data represent the health literacy on the subjects implicating people’s skills and knowledge to gain and to use this information accordingly, which is critically valuable amidst a pandemic. ^7^^,^^8^

The primary aim of this cross-sectional study was to elucidate the levels of public health literacy on COVID-19, COVID-19 vaccines and vaccinations in all regions of Indonesian (West, Central and East regions, respectively), which have yet to be analyzed largely based on specific age and sex groups. Here we assessed the respondents’ functional and critical/interactive-literacy, the individual perceptions and acceptance toward COVID-19 vaccines and vaccinations, individual perception and acceptance towards other vaccinations, individual’s belief towards health protocols and COVID-19 vaccinations safety and effectiveness, and the accessibility and usage of internet and social media to gain information related to COVID-19; to determine whether there have been gaps in the health literacy levels between groups and significant relations with the sociodemographic characteristics.

## Methods

This study was conducted in compliance with the Declaration of Helsinki (revised 2013 edition), the CHERRIES (Checklist for Reporting Results of Internet E-Survey), and STROBE (Strengthening the Reporting of Observational Studies in Epidemiology) guidelines. ^9^^–^^11^ The principal objective of this cross-sectional study was to determine the levels of health literacy about COVID-19 vaccines and vaccinations (Vaccines and Vaccinations literacy-VL) in the Indonesian adult general population via a rapid survey distributed via the internet. Another objective was to assess the perceptions of the respondents about current adult immunization and beliefs about vaccinations in general, whilst analyzing their ability to seek and use information from the internet and social media; also to study any correlations of these variables with VL levels.

### Data collection

We conducted an anonymous online survey in which participants could select to take part or not. The questionnaire was built, administered, and collected via “ SurveyPlanet Pro (Survey Planet, LLC)”, an electronic platform that creates web-based surveys that can be shared through other online services, including chatting applications, email messages, and web pages. A non-probability sampling method was adopted to spread the survey URL as a web-link collector that respondents could access and send their answers. The respondents were chosen by 10 coordinators of the survey based on the ownership of a valid email address (which was used to prevent multiple attempts to fill the survey by setting it accordingly in the Survey Planet), accessibility of the net, and age (older than 18 years old). They were Indonesian people with a good comprehension of Bahasa Indonesia who reside in five main islands in Indonesia that have been highly affected by COVID-19, including Java, Kalimantan, Sumatra, Sulawesi, and Papua regions. Respondents were free to complete the questionnaire and were asked to forward the link to others if they fulfilled the above inclusion criteria. No other exclusion criteria were adopted. Respondents were asked to provide honest answers and have been given informed consent and consent for information for both study participation and publication of the survey results prior to completing the survey. All respondents understood and agreed to the informed consent and consent for information of study participation and publication of the results indicated by clicking the “Yes” and “Next” button to proceed to the survey items. The survey could be accessed and answered via PC, tablet, or smartphone.

The items in the questionnaire were adapted and translated by three medical doctors, one of them is a native speaker, from a study by Biasio *et al.* ^12^ All items had been pretested on the total of 233 respondents who passed the inclusion and exclusion criteria prior to actual study. The initial ace validity and reliability tests showed that all items were valid and reliable, with internal consistency after test-retest and inter-rater analysis (r=0.131; Cronbach’s alpha>0.60). The questionnaire was comprised of sections i.e., the respondents were provided with the rationale and scope of this study with the informed consent and consent for information of the study participation and publication of the study results for scientific purposes. By clicking the agreement button, they would go to the following seven sections composed of (1) nine questions on the sociodemographic data (age groups, sex, last formal education levels, occupation, monthly family income, geographical residence, financial difficulty during pandemic, history of positive confirmation of respondent and/or family members, comorbidity); (2) three questions to assess functional VL with a 3 point Likert scale (1 = often, 2 = sometimes, 3 = never) that measured respondents semantic and language comprehension (Extended data) ^13^; (3) a 3 point Likert scale (1 = never, 2 = sometimes, 3 = often) of 6 questions to assess the cognitive of the respondents via the interactive-critical VL items (Extended data) ^13^; (4) eight questions to assess individual perceptions and acceptance toward COVID-19 vaccines and vaccinations; (5) four questions to assess individual perception and acceptance towards other vaccinations i.e. influenza; (6) three questions to assess individuals’ belief towards health protocols and COVID-19 vaccinations safety and effectiveness; and (7) three questions to assess the accessibility and usage of net and social media to gain information related to COVID-19. The adapted two questions from a self-reported questionnaire for adulthood vaccinations prepared on the Ishikawa test for chronic non-communicable diseases that has been validated for content and construct were used to evaluate the VL levels. Three items of the questionnaire were directed to evaluate the functional VL, and six questions evaluated the interactive-critical VL according to Nutbeam’s definition. ^12^^,^^14^ The full questionnaire can be found in the Extended data. ^13^

### Ethical considerations

Ethical approval for the current study was granted by the Health Research Ethics Committee (KEPK), Faculty of Medicine, Universitas Airlangga, Indonesia (no. 145/EC/KEPK/FKUA/2021). Participants gave their informed written consent for both study participation and publication of the survey results prior to completing the survey.

### Data analysis

The score was calculated from the mean value of the answers to each scale (range 1 to 3), a higher value standing for a higher VL level. In the previous studies, these variables were treated as numerical, where comparable tools were adopted. ^12^ The SPSS software version 17.0 was used for statistical analysis, by means of descriptive tables summarizing percentages, means, standard deviations (SD), confidence intervals (CI); also, medians and non-parametric tests, as data did not homogenous and follow a normal distribution (see the Results section). The relationship between the VL scales with other ordinal/numerical variables was analyzed using Spearman's correlation test; Chi-squared, Kruskal-Wallis, and Mann-Whitney tests were used for categorical and comparison the ratio/ordinal variables, respectively. The internal consistency of the VL scales was calculated via Cronbach’s alpha coefficient; the functional- and interactive/critical-VL items inter-relations and the analysis of factors/underlying components and each question's load on these factors were identified using the Principal Component Analysis (PCA). For each analysis, an alpha level=.05 was considered significant. ^12^

## Results

**Figure 1.**
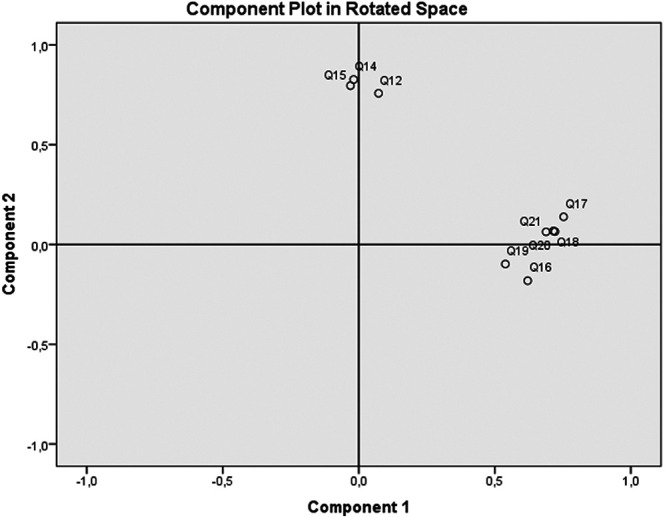
Principal Component Analysis (PCA): circle correlation between questions and components after Varimax rotation. Projection of functional VL questions (Q12, Q14, Q15) and interactive-critical VL questions (Q16-Q21) on two components (Factor 1 and Factor 2). Variables that are close to each other are significantly positively correlated.

A total of 1,143 answers were collected during the 12 weeks, starting September 2, 2021, mainly via social media and email. Most of the participants (n=512; 44.79%) answered during the second week. From Table 1, it was shown that the Q12, Q14, Q15 (questions to identify functional VL) had a strong effect on principal component 2; whilst Q16-Q21 (questions to identify interactive' critical-VL) showed a moderate to strong effect on the principal component 1. Responses to functional- and interactive/critical-VL questions exhibited good/acceptable internal consistency (Cronbach’s alpha=0.817 and 0.699, respectively), lowest values observed were 0.806 for functional scale and 0.640 for the interactive-critical scale. PCA analysis demonstrated two components accounting for 52.45% of the total variability. A varimax rotation was applied to determine relationship between items and showed that all functional VL questions were affluent on one component, whereas all interactive-critical questions were sided on the other component. The two distinguished factors loaded on the questions in each component could be discerned as predicted, i.e., close relation was observed between the questions inside each of the functional scale and of the interactive-critical one ( Figure 1, Table 1).

**Table 1.** Principal Component Analysis (PCA): correlation between questions and components (factors) after Varimax rotation. The functional vaccine literacy questions (Q12, Q14, Q15) and the interactive/critical COVID-19 vaccination literacy questions contain two components (Factor 1 and Factor 2); the value in bold corresponds to each variable with the factor with the greatest correlation.

Rotated Component Matrix ^a^		
	Components	
	1	2
Q12	.073	**.758**
Q14	-.019	**.827**
Q15	-.031	**.796**
Q16	**.621**	-.182
Q17	**.753**	.138
Q18	**.715**	.068
Q19	**.539**	-.098
Q20	**.721**	.065
Q21	**.688**	.063

Approximately 60% of respondents were females (n=686) and the rests were males (n=457). Most respondents were 18-30 years (57%, n=652), 36.2% were 31-50 years (n=414), 4.5% were 51-59 years (n=51) and only 2.3% were above 59 years (n=26). The education levels were mostly at the secondary stage (52.6%, n=601), 34.4% were at the primary level (n=393), 10.6% (n=121) were at the tertiary level, and the rests were either at the lesser degree or others. The university students were the predominant respondents (40.2%, n=460), followed by employees of the private sectors (23.9%, n=273), the civil servants (13.6%, n=155), entrepreneurs (6.6%, n=75) and others (15.7%, n=180). Respondents were mostly from Western-Indonesia (89%, n=1017), whilst only 8.6% (n=98) and 2.4% (n=28) were from the Central and Eastern-Indonesia. Most of respondents had a middle average of family income (34.7%, n=397 and 30.5%, n=349), 9.7 % (n=225) and 12.5% (n=143) had lower income, and 2.5% had a high income (n=29). About 57% (n=652) of all respondents had financial difficulty during the pandemic and 43% had no financial problem (n=491). There were 65.9% (n=753) respondents claimed that they or their household members had not been contracted COVID-19, whilst 34.1% (n=390) had been positively confirmed ( Table 2).

**Table 2.** Demographic characteristics of the respondents.

	n (%)
**Total population**	1143 (100)
Male	457 (40)
Female	686 (60)
**Age group**	
18–30	652 (57.0)
31–50	414 (36.2)
51–59	51 (4.5)
>59	26 (2.3)
**Education**	
Elementary/Junior High School	10 (.9)
Senior High School	393 (34.4)
Diploma/Bachelor	601 (52.6)
Master/Doctoral	121 (10.6)
Others	18 (1.6)
**Occupational status**	
University student	460 (40.2)
Entrepreneur (Self-employment)	75 (6.6)
Private sector	273 (23.9)
Civil servant	155 (13.6)
Other	180 (15.7)
**Residence region**	
Western Indonesia (Sumatera, Kalimantan, Java)	1017 (89.0)
Central Indonesia (Bali, West Nusa Tenggara, Sulawesi)	98 (8.6)
Eastern Indonesia (East Nusa Tenggara, Maluku, Papua)	28 (2.4)
**Average monthly expenditure in Rupiah**	
<1.416.000	143 (12.5)
1.416.000-2.128.000	225 (19.7)
>2.128.000-4.800.000	397 (34.7)
>4.800.000-24.000.000	349 (30.5)
>24.000.000	29 (2.5)
**Financial difficulties due to the COVID-19 pandemic**	
No	491 (43.0)
Yes	652 (57.0)
**Positive confirmation for COVID-19 (respondent/household members)**	
No	753 (65.9)
Yes	390 (34.1)

Most of respondents claimed that they had no comorbidities (81.7%, n=934), 9.9% (n=113) stated that they do not know if they had comorbidities; the rests stated they had one or more comorbidity i.e. respiratory-related diseases (3.4%, n=39), controlled-hypertension (3.1%, n=36), 1.6% stated to have uncontrolled-hypertension (n=18), controlled-T2DM (1.3%, n=15), uncontrolled-T2DM (0.7%, n=8), autoimmune diseases (3.4%, n=39), under-treatment-cancer (0.6%, n=7), untreated cancer (0.4%, n=5), neurological diseases (1%, n=12), 0.3% with mental illness (n=3), 0.3% (n=4) with congenital diseases and the rests claimed to have hypercholesterolemia, hyper-uremia, heart diseases, etc. (3.7%, n=43) ( Table 3).

**Table 3.** Comorbidity stated by the respondents.

Comorbidity	n (%)
No comorbidity	934 (81.7)
Not know	113 (9.9)
Cancer, under treatment	7 (.6)
Cancer, not yet/not treated	5 (.4)
Hypertension, taking medication	36 (3.1)
Hypertension, not yet/not taking medication	18 (1.6)
Diabetes, taking medication	15 (1.3)
Diabetes, not yet/not taking medication	8 (.7)
Autoimmune diseases (SLE, myasthenia gravis, rheumatoid arthritis, etc.)	12 (1.0)
Respiratory diseases (asthma, tuberculosis, chronic obstructive pulmonary disease, etc.)	39 (3.4)
Neurological diseases (stroke, Parkinson disease, Alzheimer, etc.)	12 (1.0)
Mental illness	3 (.3)
Congenital diseases	4 (.3)
Others (hypercholesterolemia, hyperuricemia, heart diseases, etc.)	43 (3.7)

There were 63.8% of respondents often use internet to search for information on COVID-19 and/or COVID-19 vaccinations (n=729), 33.2% were sometimes (n=380) and 3% never used it (n=34). Approximately 62.4% of respondents have social media account and had been using it often to engage to these topics (n=713), 33.2% (n=379) only sometimes, whilst 4.5% (n=51) never. About 74.4% (n=850) respondents sometimes believed these information, 19.1% (n=218) often, and 6.6% (n=75) had never been ( Table 4).

**Table 4.** Internet and social media use by the respondents.

	n (%)
**Do you use the internet (including Google, YouTube, etc.) to search for information regarding COVID-19 and/or COVID-19 vaccinations?**	
Often	729 (63.8)
Sometimes	380 (33.2)
Never	34 (3.0)
**Do you have social media accounts (Twitter, Facebook, Instagram, WhatsApp, Line, Telegram, TikTok, etc.) and get information regarding COVID-19 and/or COVID-19 vaccinations through these social media platforms?**	
Often	713 (62.4)
Sometimes	379 (33.2)
Never	51 (4.5)
**Do you trust the information you get through these social media (Twitter, Facebook, Instagram, WhatsApp, Line, Telegram, TikTok, etc.) regarding COVID-19 and/or COVID-19 vaccination?**	
Often	218 (19.1)
Sometimes	850 (74.4)
Never	75 (6.6)

### The score of vaccines literacy (VL)

The mean score of functional-VL was 2.41±0.49 (median 2.33); whilst the interactive/critical-VL mean score was 2.38±0.43 (median 2.5), out of a maximum of 3 ( Table 5). The functional-VL in males and females were 2.4±0.5 and 2.41±0.49 (no significant differences); and the interactive/critical-VL was lower in males than females that were 2.33± 0.46 and 2.4±0.41 (p<0.05, two-way Mann-Whitney).

**Table 5.** Functional vaccine literacy scores and interactive/critical COVID-19 vaccination literacy scores of the total population, men, and women.

	Functional vaccine literacy score mean (SD) [95% CI]	Interactive/critical vaccine literacy score mean (SD) [95% CI]	Functional vaccine literacy median (25-75 P)	Interactive/critical vaccine literacy median (25-75 P)
**Total population N=1143**	2.41 (0.49) [2.38-2.4]	2.38 (0.43) [2.35-2.40]	2.33 (2.0-2.67)	2.50 (2.17-2.67)
**Males N=457**	2.40 (0.5)	2.33 (0.46)	2.33 (2.0-3.0)	2.33 (2.0-2.67) *
**Females N=686**	2.41 (0.49)	2.40 (0.41)	2.33 (2.0-2.67)	2.50 (2.17-2.67) *

* P<0.05, Mann-Whitney.

### Perception and acceptance towards COVID-19 vaccines and vaccinations

For the question “Do you think that is a possibility to have safe and effective COVID-19 vaccines?” 848 answered “yes” (74.2%), 228 answered “don’t know” (19.9%) and 67 (5.9%) answered “no”; significant differences were observed related with the functional-VL (p=0.008), with the interactive/critical-VL (p<0.001), and with the sexes (p=0.04), but not significantly different between age groups (p=0.18).

For the question “Are you willing to get COVID-19 vaccinations?” 1092 answered yes (95.5%), 33 answered “don’t know” (2.9%) and 18 (1.6%) answered “no”; significant differences were observed only with the interactive/critical-VL (p<0.01).

For the question “Do you think the government can successfully reach the vaccinations target evenly in all provinces?” 770 answered “yes” (67.4%), 233 answered “don’t know” (20.4%) and 140 (12.2%) answered “no”; a significant difference was observed only with the interactive/critical-VL (p<0.01).

For the question of “Are you willing to pay for COVID-19 vaccinations?” 454 answered “yes” (39.7%), 143 answered “don’t know” (12.5%) and 546 (47.8%) answered “no”; significant differences were observed related with the functional-VL (p=0.008), with the interactive/critical-VL (p<0.001), and with the sexes (p=0.04), but not significantly different between age groups (p=0.18). Significant differences were observed in the critical/interactive-VL (p<0.01) and between the sexes (p=0.045).

For the question “Do you think school-age children must get COVID-19 vaccinations?” 970 answered “yes” (84.9%), 70 answered “don’t know” (6.1%) and 103 (9%) answered “no”; significant differences were observed related with the functional-VL (p=0.008), with the interactive/critical-VL (p<0.001), and with the sexes (p=0.04), but not significantly different between age groups (p=0.18). Significant differences were found between the interactive/critical-VL (p<0.01) and between the age groups (p<0.01).

For the question “Do you think certain brands of COVID-19 vaccines are safer and more effective compared to the other brands?” 514 answered “yes” (45%), 419 answered “don’t know” (36.7%) and 210 (18.4%) answered “no”; significant differences were observed related with the functional-VL (p<0.01), with the interactive/critical-VL (p<0.001), and with the sexes (p<0.01), but not significantly different between age groups (p=0.937).

For the question of “Do you have more assurance towards certain brands of COVID-19 vaccines based on the information you obtain from the internet?” 537 answered “yes” (47%), 240 answered “don’t know” (21%) and 366 (32%) answered “no”; significant differences were observed related with the functional-VL (p=0.008), with the interactive/critical-VL (p<0.001), and with the sexes (p=0.04), but not significantly different between age groups (p=0.34).

For the question of “Do you have more assurance towards certain brands of COVID-19 vaccines based on the information you obtain from the authority?” 747 answered “yes” (65.4%), 197 answered “don’t know” (17.2%) and 199 (17.4%) answered “no”; significant differences were observed related both with the functional-VL (p<0.01) and with the interactive/critical-VL (p<0.001) ( Table 6).

**Table 6.** Perceptions and acceptance of COVID-19 vaccines and vaccinations and their association with vaccine literacy (VL) scores, age groups, and genders.

				Functional VL	Interactive/critical VL	Age group	Gender
	Answer	N	%	P, Kruskal-Wallis	P, Kruskal-Wallis	P, chi-squared	P, chi-squared
**Perception and Acceptance of COVID-19 vaccination**							
Do you think that is a possibility to have a safe and effective COVID-19 vaccines?	Yes	848	74.2	**0.008**	**0.000**	.180	**.040**
	No	67	5.9				
	Don’t know	228	19.9				
Are you willing to get COVID-19 vaccination?	Yes	1092	95.5	0.190	**0.000**	.377	.838
	No	18	1.6				
	Don’t know	33	2.9				
Do you think the government can successfully reach the vaccination target evenly in all provinces?	Yes	770	67.4	0.452	**0.000**	.152	.954
	No	140	12.2				
	Don’t know	233	20.4				
Are you willing to pay for COVID-19 vaccination?	Yes	454	39.7	0.088	**0.000**	.222	**.045**
	No	546	47.8				
	Don’t know	143	12.5				
Do you think school-age children must get COVID-19 vaccination?	Yes	970	84.9	0.074	**0.000**	**.001**	.334
	No	103	9.0				
	Don’t know	70	6.1				
Do you think certain brand of COVID-19 vaccine are safer and more effective compared to the other brands?	Yes	514	45.0	**0.000**	**0.000**	.937	. **001**
	No	210	18.4				
	Don’t know	419	36.7				
Do you have more assurance towards certain brand of COVID-19 vaccine based on the information you obtain from the internet?	Yes	537	47.0	0.467	**0.000**	.340	**.044**
	No	366	32.0				
	Don’t know	240	21.0				
Do you have more assurance towards certain brand of COVID-19 vaccine based on the information you obtain from the authority?	Yes	747	65.4	**0.001**	**0.000**	.638	.082
	No	199	17.4				
	Don’t know	197	17.2				
**Individual perception and attitude towards other vaccines**							
Have you been vaccinated other than the COVID-19 vaccine before? (eg: Measles, DPT, Polio, Meningitis, Hepatitis, BCG, HPV, Influenza, Pneumonia, etc.)	Yes	768	67.2	0.624	**0.000**	**.012**	**.028**
	No	153	13.4				
	Don’t know	222	19.4				
Have you been vaccinated against influenza before?	Yes	198	17.3	0.728	**0.000**	.645	**.000**
	No	623	54.5				
	Don’t know	322	28.2				
Do you plan to get vaccinated against influenza and/pneumonia this year and/next?	Yes	205	17.9	0.491	**0.000**	.176	**.001**
	No	441	38.6				
	Don’t know	497	43.5				
Are you planning to get any vaccinations other than the COVID-19 vaccine this year? (eg: Measles, DPT, Polio, Meningitis, Hepatitis, BCG, HPV, Influenza, Pneumonia, etc.)	Yes	120	10.5	0.321	**0.000**	**.030**	**.000**
	No	468	40.9				
	Don’t know	555	48.6				

All bold numbers of P significant (P<0.05).

Perception and acceptance towards the COVID-19 vaccines and vaccinations were mostly positive, with affirmative responses between approximately 74.2% and 95.5%. However, most respondents had higher hesitancy to pay for the vaccines and vaccinations (47.8%) and 454 respondents were willing-to-pay (39.7%). The acceptance on the information regarding these topics was higher when coming from the authority than on the internet (65.4% *vs.* 47%). Acceptance toward COVID-19 vaccinations was quite high, which were significantly correlated with both functional- and interactive/critical-VL (r=0.112, p<0.001 and r=0.264, p<0.01); but not significantly related with either education level or the age group (r=-0.04, p=0.898 and r=-0.31, p=0.299, respectively).

### Belief and personal value toward COVID-19 vaccines and vaccinations

Only a minority of respondents agreed completely (Likert score 3/no) with the statements: “I am not favourable to COVID-19 vaccines because they are unsafe and/or ineffective” (6.7%, n=76), “There is no need to get COVID-19 vaccinations because natural immunity already exists” (6%, n=69), whilst complete disagreement with statement: “Health protocols i.e. wearing a mask in public, physical distancing and washing hands are important things to do, in addition to COVID-19 vaccinations can help to lower the morbidity” was only 3% (n=35). On the other hand, most respondents were in complete disagreement with the first two statements (80.1%, n=914 and 84.7%, n=968, respectively) and mostly agreed with the last statement above (94.1%, n=1076). Answers with “don’t know” on all of these statements were 13.2% (n=151), 9.3% (n=106) and 2.8% (n=32), respectively.

There were significant correlations between each of these three statements response with the interactive/critical-VL (r= 0.264, p<0.01; r=0.192, p<0.01; r=0.135, p<0.01, respectively). Significant correlations were observed between each of the first two statements response with the functional-VL (r=0.112, p<0.01 and r=0.103, p<0.01) ( Table 7).

**Table 7.** Spearman rank correlation coefficient (r) and the level of significance between the ordinal and ordinal/numeric variables observed in the survey (significant p values in bold).

		Education	Age group	Monthly family expenditure	Agreement to statement 1 ^*^	Agreement to statement 2 ^**^	Agreement to statement 3 ^***^
**Functional VL**	Correlation coefficient	-0.022	-.068 ^*^	.046	.112 ^**^	.103 ^**^	.050
	Significance (p)	.448	**.022**	.116	**.000**	**.000**	.092
**Interactive/critical VL**	Correlation coefficient	.109 ^**^	-.062 ^*^	.162 **	.264 ^**^	.192 ^**^	.135 ^**^
	Significance (p)	**.000**	**.037**	**.000**	**.000**	**.000**	**.000**
**Education**	Correlation coefficient		.389 ^**^	.195 **	-.004	-.009	.041
	Significance (p)	.	**.000**	**.000**	.898	.755	.161
**Age group**	Correlation coefficient		1.000	.271 **	-.031	-.049	.048
	Significance (p)		.	**.000**	.299	.098	.102
**Monthly family expenditure**	Correlation coefficient				.120 **	.073 *	.076 *
	Significance (p)				**.000**	**.013**	**.010**

All bold numbers of P significant (P<0.05).

^*^ Statement 1: I don't want to be vaccinated because the COVID-19 vaccine is not safe and/or ineffective.

^**^ Statement 2: I don't need to be vaccinated against COVID-19 because I already have the body's natural immunity.

^***^ Statement 3: Health protocols such as wearing masks, washing hands and keeping a distance and avoiding crowds can reduce the transmission of COVID-19 in addition to COVID-19 vaccination.

### Behaviours toward other vaccinations

In Table 6, approximately 67.2% (n=768) of participants self-reported that they had been vaccinated the previous years for at least one of these vaccinations: tetanus, pneumonia, diphtheria, pertussis, polio, measles, TBC, HPV, meningitis, hepatitis; significant differences were found in the interactive/critical-VL (p<0.01), between age groups (0.012) and sexes (p=0.028). Approximately 10.5% (n=120) respondents stated their intention to get one of these vaccinations again in the next season; significant differences were observed in the interactive/critical-VL (p<0.01), between age groups (p=0.03) and sexes (p<0.01).

About 17.3% (n=198) respondents have been vaccinated for influenza; 17.9% (n=205) claimed they intend to receive another influenza and/or pneumonia vaccinations during the next season. Significant differences were found in the interactive/critical-VL (p<0.01) and between sexes (p<0.01).

### Correlation of VL with other variables

**Figure 2.**
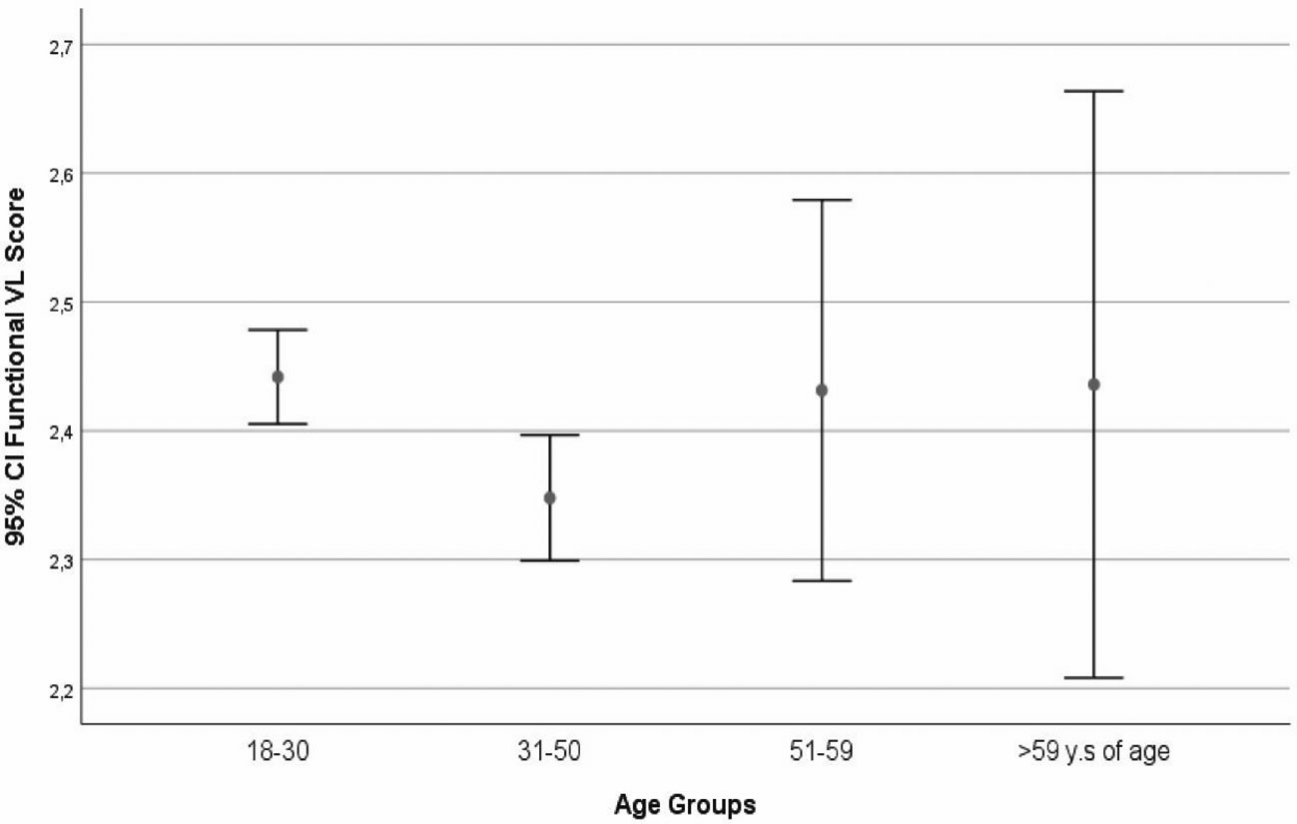
Observed functional Vaccines Literacy (VL) scores, visualized as mean and 95% CI (error bars), according to age groups.

**Figure 3.**
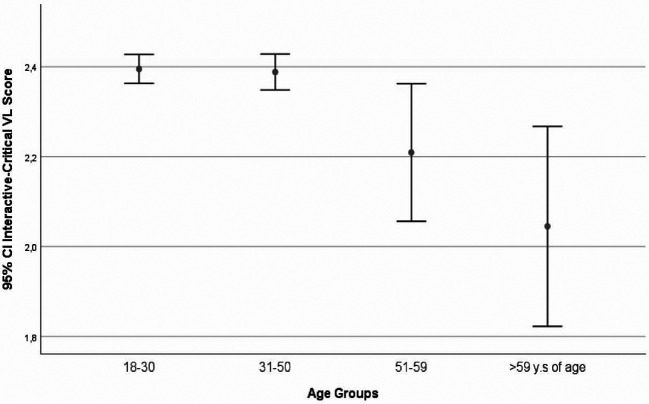
Observed interactive-critical Vaccines Literacy (VL) scores, visualized as mean and 95% CI (error bars), according to age groups.

The correlations between functional-VL with either education level or the age group were negative, with significance showed with the latter (r=-0.022, p=0.448 and r=-0.68, p=0.022). The interactive/critical-VL had significant correlations with both education level and the age group (r=0.109, p≤0.001 and r=-0.062, p=0.037). Significant correlations were found between the functional-VL and the interactive/critical-VL (r=0. 084, p=0.005) ( Figures 2- 3).

## Discussion

Coronavirus disease 2019 (COVID-19) has become a worldwide challenge that has affected the health systems of many countries, including Indonesia. ^15^^,^^17^ SARS-CoV-2 mutated virus produces variants with dynamic responses to available vaccines. ^18^ Although research is still emerging, the reported data show that people with chronic and comorbid illnesses are highly susceptible to COVID-19, ^19^^,^^20^ and experience higher levels of disease severity when they get infected. ^21^^–^^23^ Antiviral treatments and vaccines that are proven to be fully effective against viral mutations are under ongoing development. ^24^^,^^25^ Various scientific studies continue to grow every day; however, the research results are still mixed and often raise questions in the community. ^26^^,^^27^ The need to master the skill to understand, access, and act accordingly based on valid health information (health literacy) is emerging to be a vital psychosocial factor of health outcomes, determined by sociodemographic characteristics i.e., age, ethnicity, and economic levels. ^7^^,^^28^ The proposed effective way to contain this problem is to build good health literacy about COVID-19 and COVID-19 vaccinations, including via this online survey, as the public can contribute to the preventive and promotive measurement by protecting themselves while protecting others, to halt the spread of COVID-19. ^27^^,^^29^ As a result, health literacy is crucial because it becomes the centre value in filtering information about COVID-19 and COVID-19 vaccinations. Access to this information also plays a vital role in the level of health literacy, ^30^^–^^32^ although hoaxes can produce misunderstandings. ^33^^,^^34^

As an archipelago, Indonesia has a geographical disparity with cultural diversity that influences values and behaviour in facing external challenges i.e., COVID-19. ^15^^,^^35^ In addition, socioeconomic and educational levels determine literacy. ^36^ Individual compliance to health protocols, including physical distancing, voluntary screening tests, self-isolation if infected, face mask use in public places, and hand sanitation are various. ^16^^,^^37^ Together with the national vaccinations program, the infection rate has decreased by more than 90% since July 2021 when the Delta variant began to strike. ^38^ Restriction measures are applied by requiring returning travellers to do a quarantine, restricting public recreational places, non-essential sectors, offices, and schools to implement emergency micro-based social activity restrictions (PPKM) since 2020. ^39^ However, the disparity of engagement levels in these efforts might produce hotspots with additional waves of mutated-virus infection, certain clusters might be suffering more severely affected by COVID-19. ^40^^,^^41^

This rapid-on-line survey was responded predominantly by female participants and the age group between 18-50 years. They mostly graduated from the primary and secondary education levels, and this was presented in the predominant occupation of the respondents were university students, while private and public employees came next. The lack of direct promotion and distribution of the online survey during the pandemic might be the reason of the predominant respondents were from Java, Sumatra, and Kalimantan than from other regions i.e. Sulawesi, Maluku, Bali, Nusa Tenggara, and Papua. ^42^ However, this could serve the purpose of this study to capture the data from where the highest incidence of COVID-19 was reported from that was in Western Indonesia. The monthly family income was mostly at between 2-24 million (IDR), which is classified as a middle-class economy, this could be affected by the pandemic as stated by more than half of respondents who also claimed that this pandemic had struck at least one of their household family members.

Approximately more than 80% of all respondents stated that they were free from any comorbidity i.e., respiratory-related diseases (asthma, COPD), hypertension, cancer, T2DM, psycho-neurology disorders, congenital abnormality, metabolic syndrome, and other pathological conditions, although about 10% was aware of these comorbidities. During the pandemic, an increasing number of people relied on the internet and social media to obtain information about COVID-19. In our study, we found that all respondents used the internet to seek information regarding COVID-19 and COVID-19 vaccinations, amongst these, they used at least one social media actively, with a high percentage of respondents sometimes believing the validity of this information (74.4%). ^43^^,^^44^

From the maximum number of the Likert scale of the vaccines literacy (VL), we observed high scores in both functional- and interactive/critical-VL where these were quite in a balance between sexes in the prior VL and higher in females for the latter. Perception and acceptance toward COVID-19 vaccines and vaccinations were generally positive shown from the predominant good responses on the safety and effectiveness of the vaccines and from the willingness to be vaccinated. However, when asked to pay for the vaccinations, almost half of the respondents disagreed (47.8%), 20.4% were still in doubt, whilst the other 39.7% agreed. More than 80% of respondents agreed that school-aged children must be vaccinated against COVID-19; 45% agreed that certain vaccine brands have more safety and effectiveness than the others (mostly due to the RNA-based *vs.* weakened- and/or killed virus-based vaccines) whilst about 47% respondents felt more self-assurance after they read the information on certain vaccines via internet. The acceptance on the information regarding these topics was higher when coming from the authority than the internet (65.4% *vs.* 47%); acceptance toward COVID-19 vaccinations was significantly correlated with both functional- and interactive/critical-VL but not significantly related to either education level or the age group. These data showed us that even though lots of people seek information using the internet, whilst might decrease their anxiety about the unknown part of COVID-19 vaccines and vaccinations, the validity of the information is questionable and they preferred the information from the authority. ^45^^,^^46^ Here, perception and acceptance of COVID-19 vaccines and vaccinations of Indonesian people were shown to have more strong correlation with the interactive/critical-VL rather than the functional-VL or other socio-demography characteristics i.e., sex and age group. ^38^

The disagreement of getting COVID-19 vaccinations due to its safety and/or effectiveness also due to the belief in natural immunity was quite low, whilst most respondents agreed at the value of conducting health protocols regardless of the vaccination states. The first two statements' perception and belief were significantly correlated with the functional VL and the interactive/critical VL, while the last statement was only significantly correlated with the functional VL. Arguably, the values play a vital role in facing the pandemic and speeding up the national vaccinations coverage that has started since the beginning of 2021 in Indonesia. ^47^^,^^48^ More respondents had been vaccinated against various infectious diseases, although only about 17.3% had been vaccinated for the influenza. Only about 10-18% of respondents claimed their intention to have these vaccinations again in the next season; these were significantly correlated with the interactive/ critical-VL and sex. These data might show us that most healthy persons had relatively low literacy on this topic; these vaccinations have more frequently done amongst school-age children than adults in Indonesia. Previous study on the consumption of supplements during the COVID-19 pandemic in Indonesia found that these behaviours was influenced by education, age, family income, and family expense. ^49^ We observed that both VL were significantly correlated with the age group, the highest was found in the 18-50 years age groups; whilst the interactive/critical-VL had a parallel and significant correlation with the education level.

To the best of our knowledge, this study is the first to analyze health literacy on COVID-19 and COVID-19 vaccines and vaccinations. The current study was carried out in a period between the first and the second dose of the COVID-19 vaccinations program was running. The results would be valuable for implementing boosters involving large populations where further studies are called. The VL has been used to show health literacy on various studies. Here it represented people's acceptance, perception and attitude towards COVID-19 and its vaccinations. The hesitance to the vaccinations was shown due to i.e., personal belief and knowledge, willingness-to-pay, accessibility, and the influence from the community and the authority. A vast amount of information on COVID-19 potentially confused people to the extent of believing or rejecting all news related to the disease. ^50^ Health literacy can assist people in filtering and finding correct information and then using these accordingly; including logically reason the vaccinations and health protocols. ^12^^,^^51^^,^^52^ Health education must be adjusted according to local wisdom and condition to meet the needs of diverse clusters hence could bridge the gap in the community with various health literacy. ^6^^,^^50^

This study adapted the convenience sampling in which university students were the predominant respondents. This was due to the practical reasons that these respondents are the most likely ones who regularly use the internet and social media and were familiar with the online survey. Another limitation is that the respondent’s demography was mostly from Java and least from the eastern part of Indonesia. Self-claimed responses might affect the generalization of study results. However, the PCA of both VL scales showed good internal consistency and component loading.

Furthermore, findings from the current study are valuable due to its topics and the relatively broad range of respondents in terms of education levels and family income can represent most part of the population; levels of VL and their associations to various independents variables were comparable to other similar studies, validated using direct interview methods. Uneven levels of health literacy are an adverse situation that might slow the successful rate against the pandemic. Further studies are needed especially on the comprehension of the importance of vaccinations and health protocols that can help people to adapt to the new normal era.

## Conclusions

A self-stated online survey conducted in the current study can provide reliable data on the level of health literacy of quite a large sample population distributed in different islands. The findings of this study could help community and decision-maker parties prepare communication and action strategies to cope with the pandemic. The VL levels of all respondents showed strong relationships with the individual perception, belief, and acceptance toward vaccinations and health protocols. Valid information regarding COVID-19 from authority are called for and could help the public to act accordingly.

## Data Availability

Dryad: Survey Data of the Health Literacy on COVID-19 and COVID-19 Vaccination in Indonesia, https://doi.org/10.5061/dryad.2fqz612sg. ^13^ This project contains the following underlying data:
- Full response data.xlsx Dryad: Survey Data of the Health Literacy on COVID-19 and COVID-19 Vaccination in Indonesia, https://doi.org/10.5061/dryad.2fqz612sg. ^13^ This project contains the following extended data:
- English translation of the full questionnaire.pdf Data are available under the terms of the Creative Commons Zero “No rights reserved” data waiver (CC0 1.0 Public domain dedication).
