# Supplementary material for: Health literacy on COVID-19 and COVID-19 vaccinations in Indonesia
Source: F1000Res. 2022 Dec 23;11:1296. Originally published 2022 Nov 11. [Version 2] doi: 10.12688/f1000research.125551.2 (PMC9811029; doi:10.12688/f1000research.125551.2)
Supplement: Peer review history

# Peer review: Reviewer response for version 2

**DOI:** 10.5256/f1000research.142377.r158550

**Published:** 2023-01-03

**Authors:** Puspa Sari

**Affiliations:** 1 Faculty of Medicine, Padjadjaran University, Bandung, Indonesia

The authors have made changes to all revisions, and I have no further comments.

Is the work clearly and accurately presented and does it cite the current literature?

Yes

If applicable, is the statistical analysis and its interpretation appropriate?

Yes

Are all the source data underlying the results available to ensure full reproducibility?

Yes

Is the study design appropriate and is the work technically sound?

Yes

Are the conclusions drawn adequately supported by the results?

Yes

Are sufficient details of methods and analysis provided to allow replication by others?

Partly

Reviewer Expertise:

Public Health, Midwifery, Adolescent and Women Reproductive Health

I confirm that I have read this submission and believe that I have an appropriate level of expertise to confirm that it is of an acceptable scientific standard.

# Peer review: Reviewer response for version 1

**DOI:** 10.5256/f1000research.137870.r155592

**Published:** 2022-12-06

**Authors:** Puspa Sari

**Affiliations:** 1 Faculty of Medicine, Padjadjaran University, Bandung, Indonesia

In this study the Authors investigated Health literacy on COVID-19 and COVID-19 vaccinations in Indonesia. This is an interesting and relevant article according to the current condition of the covid pandemic, and this will support the efforts of health workers to increase public knowledge about covid 19 and covid 19 vaccinations. However, I have the following comments and questions of the manuscript:

**Abstract:**

 The title and abstract cover the main aspect of the work.

**Introduction:**

 Background and study-related information are provided in the introduction. But the author may clarify whether earlier research similar to this has been done. If not, explain that this is a novelty of this research. Explain whether there are any official information sources in Indonesia that the general public can access.

**Methods:**

 Please explain the sample size. Is there no minimal sample?

**Results:**

 Provides new, helpful facts for Indonesia

**Discussion:**

 Relevant discussion. Limitations and implications are described.

Is the work clearly and accurately presented and does it cite the current literature?

Yes

If applicable, is the statistical analysis and its interpretation appropriate?

Yes

Are all the source data underlying the results available to ensure full reproducibility?

Yes

Is the study design appropriate and is the work technically sound?

Yes

Are the conclusions drawn adequately supported by the results?

Yes

Are sufficient details of methods and analysis provided to allow replication by others?

Partly

Reviewer Expertise:

Public Health

I confirm that I have read this submission and believe that I have an appropriate level of expertise to confirm that it is of an acceptable scientific standard, however I have significant reservations, as outlined above.

# Response

**Published:** 2022-12-19

**Authors:** Viskasari Kalanjati

**Affiliations:** Airlangga University, Indonesia

Puspa Sari, 

 School of Medicine and Public Health, University of Newcastle, Callaghan, Australia

 Introduction:

 Background and study-related information are provided in the introduction. But the author may clarify whether earlier research similar to this has been done. If not, explain that this is a novelty of this research. Explain whether there are any official information sources in Indonesia that the general public can access.

 Author’s response: Thank you for the insightful advice, we have added a previous study from Indonesian sample population that would explain about this in the inteoduxtion, as followed: 

 " In the study by Harisanty et al., (2021), it was reported that the digital literacy level of COVID-19 amongst 500 respondents in east Java, Indonesia was 3.69 and represented a good average level. 5 – 7  "

 Methods:

 Please explain the sample size. Is there no minimal sample?

 Author’s response: Thank you, we have added it to the method section, as followed:

 "...The minimum sample size was calculated using a sample size calculator (http://www.raosoft.com/samplesize.html) with 3% margin of error and 95% confidence level.  The minimum sample size required in this study was 1014."

# Peer review: Reviewer response for version 1

**DOI:** 10.5256/f1000research.137870.r155593

**Published:** 2022-12-01

**Authors:** Athira Nandakumar

**Affiliations:** 1 Department of Epidemiology and Preventive Medicine, Kagoshima University Graduate School of Medical and Dental Sciences, Kagoshima, Japan

**INTRODUCTION**

 The importance of public health literacy especially during a pandemic is the most relevant and the authors through this online survey study very well showed how it prevailed in Indonesian islands together with COVID-19.

**Comment: **The introduction part lacks the background or already existing health literacy information in Indonesia.

**METHODS**

1) Data Collection:

- **Comment:** availability of net should be changed to internet.
- **Question: **The study seems to be done in the major areas like Java, Kalimantan, Sumatra, Sulawesi, and Papua regions, so the participants' number might have been less, N is just 1143. What was the response rate?
- **Question: **What were the selection criteria for the respondents, and how they conformed their residence?

2) Data Analysis:

- **Question: **How the authors treated the effect of confounding factors is not clear.
- **Comment: **Probably regression analysis adjusting for potential confounding variables can be an alternative model for analysis.

3) Results:

- **Table 2, Question: **The residence area names mentioned in Table – western, eastern and central Indonesia doesn’t correspond to the 5 area names mentioned in the Introduction. Please use a uniform style in names. The table shows the main area is the western region with around 90 percent participants
- **Table 5, Comment: **Adding some simple definitions of Functional, Interactive, or critical vaccine literacy scores will be easier for the readers.
- **Table 5, Question: **Just the Median of the vaccine literacy scores makes it clear, and hence mean is not necessary. Or is it based on some analysis?
- **Table 5, Question: **Functional vaccine literacy score for the total population is 2.41. Is that a good score? Is it comparable?

4) Perception and acceptance towards COVID-19 vaccines and vaccinations:

- **Comment:** Citing Table 6 alone is sufficient to portray the purpose.

5) Correlation of VL with other variables:

- Figure 2, **Question: **Why age groups are selected; especially because 57% are in the first group, education and occupational status could have been better alternatives.
- Figure 2,3 **Comment: **Y-axis labels can be just 0,1,2,3;
- Figure 2,3 **Question: **Why the authors choose the order of categories in the X-axis is not clear.

Is the work clearly and accurately presented and does it cite the current literature?

Yes

If applicable, is the statistical analysis and its interpretation appropriate?

Yes

Are all the source data underlying the results available to ensure full reproducibility?

Yes

Is the study design appropriate and is the work technically sound?

Yes

Are the conclusions drawn adequately supported by the results?

Yes

Are sufficient details of methods and analysis provided to allow replication by others?

Yes

Reviewer Expertise:

Epidemiology and Preventive Medicine

I confirm that I have read this submission and believe that I have an appropriate level of expertise to confirm that it is of an acceptable scientific standard.

# Response

**Published:** 2022-12-19

**Authors:** Viskasari Kalanjati

**Affiliations:** Airlangga University, Indonesia

Athira Nandakumar, Department of Epidemiology and Preventive Medicine, Kagoshima University Graduate School of Medical and Dental Sciences, Kagoshima, Japan

 INTRODUCTION

 The importance of public health literacy especially during a pandemic is the most relevant and the authors through this online survey study very well showed how it prevailed in Indonesian islands together with COVID-19.

 Comment: The introduction part lacks the background or already existing health literacy information in Indonesia.

 Author comment: Thank you, we have made a revision

 METHODS

 1) Data Collection:

 Comment: availability of net should be changed to internet.

 Author’s response: Thank you, already revised

 Question: The study seems to be done in the major areas like Java, Kalimantan, Sumatra, Sulawesi, and Papua regions, so the participants' number might have been less, N is just 1143. What was the response rate?

 Author’s response: The response rate was 1143/1153 (99%). A total of 1153 respondents gave responses, however, 10 of them declined to finish the survey due to technical matters, hence the number of total respondents included in our analysis was 1143. 

 Question: What were the selection criteria for the respondents, and how they conformed their residence? 

 Author’s response: Thank you for your question, the answers of these have been detailed in the methods, as followed: ’… The respondents were chosen by 10 coordinators of the survey based on the ownership of a valid email address (which was used to prevent multiple attempts to fill the survey by setting it accordingly in the Survey Planet), accessibility to the internet, and age (older than 18 years old). They were Indonesian citizens with a good comprehension of Bahasa Indonesia who reside in the western (Sumatera, Java, and Kalimantan islands), central (Bali, West Nusa Tenggara, Sulawesi Islands), and eastern (East Nusa Tenggara, Maluku, Papua Islands) regions of Indonesia”.

 We asked all respondents to give honest answers whilst 10 in-charged surveyors confirmed the residence of each respondent personally as we did not require them to submit any personal identity card as proof due to security reasons.

 2) Data Analysis:

 Question: How the authors treated the effect of confounding factors is not clear.

 Comment: Probably regression analysis adjusting for potential confounding variables can be an alternative model for analysis

 Author’s response: Thank you for your comment. We have applied homogeneity and normality tests to control the variance and distribution of the data set whilst filtering the respondent’s background variability using strict inclusion and exclusion criteria, thus minimizing potential bias. We then applied non-parametric inferential statistics to compare the median of the variables and employed a level of significance of p < 0.05 with 95% of CI. 

 3) Results:

 Table 2, Question: The residence area names mentioned in Table – western, eastern and central Indonesia doesn’t correspond to the 5 area names mentioned in the Introduction. Please use a uniform style in names. The table shows the main area is the western region with around 90 percent participants

 Author’s response: Thank you, we have made a revision accordingly. 

 Table 5, Comment: Adding some simple definitions of Functional, Interactive, or critical vaccine literacy scores will be easier for the readers.

 Author’s response: Thank you for your comment. We have detailed the definition Functional, Interactive, or critical vaccine literacy scores in the method section in the paragraph right before the ethical consideration.

 Table 5, Question: Just the Median of the vaccine literacy scores makes it clear, and hence mean is not necessary. Or is it based on some analysis?

 Author’s response: Thank you for your comment. The comparison of means was done in the parametric data set, while the medians were compared in non-parametric data set, thus implied in Table 5. 

 Table 5, Question: Functional vaccine literacy score for the total population is 2.41. Is that a good score? Is it comparable?

 Author’s response: Thank you for your question. The Functional vaccine literacy score were assessed using three questions with a 3-point Likert scale (1 = often, 2 = sometimes, 3 = never) that measured respondents’ semantic and language comprehension; thus, 2.41 score of the total population represented that the major respondents never or only sometimes found difficulties regarding the semantic and the language when understanding COVID-19 vaccines and vaccination information.

 4) Perception and acceptance towards COVID-19 vaccines and vaccinations:

 Comment: Citing Table 6 alone is sufficient to portray the purpose.

 Author’s response: We agree, thank you very much.

 5) Correlation of VL with other variables:

 Figure 2, Question: Why age groups are selected; especially because 57% are in the first group, education and occupational status could have been better alternatives.

 Author’s response: Thank you for your comment. We have chosen analysis based on the age groups for this particular figure, however, we also detailed further analysis based on the education and monthly income comparison in Table 7. 

 Figure 2,3 Comment: Y-axis labels can be just 0,1,2,3;

 Author’s response: Thank you for your comment, we have revised accordingly. 

 Figure 2,3 Question: Why the authors choose the order of categories in the X-axis is not clear.

 Author’s response: Thank you for your comment, we have revised accordingly.
